# Yerba Maté (*Ilex paraguariensis*) Metabolic, Satiety, and Mood State Effects at Rest and during Prolonged Exercise

**DOI:** 10.3390/nu9080882

**Published:** 2017-08-15

**Authors:** Ahmad Alkhatib, Roisin Atcheson

**Affiliations:** Dasman Diabetes Institute, Dasman, Kuwait City 15462, Kuwait; Atcheson-R@email.ulster.ac.uk

**Keywords:** fat-loss, appetite, metabolism, endurance-exercise, thermogenic, psychomotor

## Abstract

Yerba Maté (YM), has become a popular herb ingested for enhancing metabolic health and weight-loss outcomes. No studies have tested the combined metabolic, satiety, and psychomotor effects of YM during exercise. We tested whether YM ingestion affects fatty acid oxidation (FAO), profile of mood state score (POMS), and subjective appetite scale (VAS), during prolonged moderate exercise. Twelve healthy active females were randomized to ingest either 2 g of YM or placebo (PLC) in a repeated-measures design. Participants rested for 120 min before performing a 30-min cycling exercise corresponding to individuals’ crossover point intensity (COP). FAO, determined using indirect calorimetry, was significantly higher during the 30-min exercise in YM vs. PLC (0.21 ± 0.07 vs. 0.17 ± 0.06 g/min, *p* < 0.05). VAS scores for hunger, prospective eating, and desire to eat were all reduced (*p* < 0.05). Whereas, POMS measures of focus, energy, and concentration were all increased (*p* < 0.05). There was no significant time-effect for any of the measured variables, nor was there any interaction effects between YM treatment and time. Combining YM intake with prolonged exercise at targeted ”fat-loss”’ intensities augments FAO and improves measures of satiety and mood state. Such positive combined metabolic, satiety, and psychomotor effects may provide an important role for designing future fat and weight-loss lifestyle interventions.

## 1. Introduction

Nutritional and exercise lifestyle strategies are primary tools for weight and fat loss to prevent major health risks and lifestyle diseases such as obesity, diabetes, and cardiovascular disease. The effectiveness of healthy lifestyle strategies can be maximized with a variety of nutritional aids in the form of functional foods and naturally available herbal ingredients [1,2].

Yerba Maté (YM), the plant *Ilex paraguariensis*, is traditionally consumed in many South American regions, but its popularity is increasing in North America, Europe, and other regions worldwide [3]. Several anti-atherogenic and weight-loss properties have been associated with the regular consumption of YM [4], including anti-oxidation, vasodilation, reduction in blood lipids, and other anti-mutagenic and anti-glycation benefits [5,6,7,8,9]. These health properties have been attributed to several naturally-present bioactive ingredients, which have been detected in YM including polyphenols and caffeoyl derivatives (caffeic acid, chlorogenic acid, 3,4-Dicaffeoylquinic acid, 4,5-Dicaffeoylquinic acid and 3,5-Dicaffeoylquinic acid), phytosterols, saponins, some amino acids, vitamins, and minerals [4,5]. Metabolic functions of YM components are thought to be responsible for reductions in serum cholesterol, serum triglycerides, and glucose concentrations; and an improved glycemic control and lipid profile in in high-fat fed mice [7], reduced body fat mass, and distribution and reduced waist to hip ratio in humans, all shown following YM ingestion [10,11].

Recent findings have also indicated that YM metabolic properties may be combined with positive psychomotor and appetite control effects, which complement the YM fat-loss promoting properties. Such effects include suppressed appetite through an increased expression of glucagon-like peptide-1 (GLP-1) and delayed gastric emptying, as seen in mice studies [12,13], and increased ghrelin up to 4.2-fold in rat models following YM ingestion [14].

A trend towards increased satiety, reduced hunger, and improved mood state has also been found using visual analogue scale in human participants who ingested YM combined with other fat-loss ingredients [15,16]. The reported psychomotor effects include improved total mood disturbance score [15], increased focus, alertness and energy, and decreased fatigue in habitual caffeine consumers [16]. Modifying behavioral factors of mood state and appetite control is considered essential for effective weight-loss lifestyle interventions [17,18,19]. Consequently, indications of the positive YM effects combined with exercise on those outcomes should be further investigated, especially given the known positive effects of exercise on mood state and mental health [20].

Along with the nutritional metabolic weight and fat-loss benefits, exercise is known to stimulate fat metabolism, and reverse associated metabolic health risks. YM effects on thermogenesis has been suggested to promote fat-loss by influencing indirect calorimetry measures such as energy expenditure (EE), fatty acid oxidation (FAO), and respiratory exchange ratio (RER) in resting healthy obese participants [21]. However, little is known about such YM metabolic effects during exercise. Our recent work has shown that YM favors FAO as a fuel source during exercise, when either ingested as a single ingredient [22] or combined with other fat-loss compounds in a multi-ingredient supplement [15]. In Alkhatib, 2014 it was found that 1 g of YM can induce an over 20% increase in FAO at the exercise intensity range of 40–70% of peak oxygen uptake (${\dot{V}O}_{2\mathrm{peak}}$), which is considered to be within the low-to-moderate exercise intensity domain [22]. This intensity range of exercise corresponds to maximal fat oxidation (Fatmax) intensity, defined as the exercise intensity where FAO becomes maximal, and the crossover point (COP), defined as the power output when the energy expenditure derived from carbohydrates (CHO) fuels predominates over that from FAO fuels [23,24]. Performing exercise at individually determined Fatmax or COP intensities has been shown to induce favorable metabolic outcomes, such as enhanced FAO ability, improved insulin sensitivity, and enhanced vascular function [25,26,27]. However, to date no study has tested whether the exercise-induced metabolic effects at those effective exercise intensities (i.e., COP intensities) could be augmented with YM ingestion.

Given our recent promising findings of YM acute effects on FAO during exercise intensities in the COP range [15,22], and the YM weight-loss postulated effects on satiety and mood state [13,22], our study is important to test whether and how YM affects FAO, satiety, and mood state during prolonged exercise at individuals’ COP intensities. This study aims to test the hypothesis that YM ingestion combined with steady state exercise at the COP intensities augments FAO, and improves the measures of satiety and mood state.

## 2. Materials and Methods 

### 2.1. Design and Participants

The study followed a double-blind repeated-measures crossover placebo-controlled design. All tests were performed at similar laboratory environmental conditions controlling for air temperature, barometric pressure, and relative humidity. The study was approved by the institution’s ethical committee. All experimental procedures were carried out in accordance with the ethical guidelines of the World Medical Association Declaration of Helsinki. All study participants provided their informed written consent and were given an explanation of the purpose of the research and experimental procedures.

Exclusion criteria were as follows. (1) History of any cardiovascular or respiratory disease, hypertension, liver or kidney disease, musculoskeletal or neuromuscular or neurological disease, autoimmune disease, cancer, peptic ulcers or anemia; (2) Taking medications, including those for heart, pulmonary, thyroid, anti-hyperlipidemic, hypoglycemic, anti-hypertensive, endocrinologic, psychotropic, neuromuscular, neurological, or androgenic conditions, as well as a family history of heart problems, high blood pressure, and/or stroke, and being pregnant or breastfeeding. (3) Consuming any ergogenic aid or above habitual caffeine consumption rate (≥200 mg/day) for at least six weeks prior to the study similarly to a previously used approach [16], based on all types of caffeinated beverages (coffee, energy drinks, soft drinks, caffeine supplements, or medications). Inclusion criteria included females, aged 18–40 years, habitually complete ≥150 min of moderate physical activity per week, BMI < 30.0 kg/m^2^. Participants were asked to specify the phase of their menstrual cycle (luteal or follicular phase). All participants were eumenorrheic and none of them were taking any oral contraceptive pill or any other forms of hormonal contraceptives. All participants refrained from taking any supplements for the duration of the study and were instructed to refrain from strenuous exercise or alcohol and caffeine consumption for at least 24 h before each test. Each exercise testing trial was performed within one week (between three and seven days) of the previous session. All participants were familiarized with the testing equipment and procedures prior to the start of the experiment.

Participants were physically active healthy women volunteers, and were recruited by convenient sampling. A total of 12 females completed the study [Mean ± SD, age = 30.8 ± 7.3 years, stature: 167.2 ± 3.9 m, body mass: 61.5 ± 2.8 kg, BMI = 22.0 ± 1.1 kg/m^2^, body fat percentage (BF%) 19.8 ± 4.2%]. Nine out of 21 participants who met the inclusion criteria failed to complete all tests, mainly due to lack of time or inability to commit for all testing sessions. Sample size calculations were based on achieving a large effect size based on the least meaningful difference induced by YM supplement on FAO in previous studies [22], and provided a power of 90% for an alpha level significance of 5%. The calculation showed 12 participants were needed.

### 2.2. Experimental Procedures and Exercise Protocols

All participants reported to the Physiology Laboratory on three separate occasions followed by 3 h fasting state in the first session, and 10–12 h overnight fasting in the second and third sessions. Each testing session (between 09:30 and 11:30 a.m.) was separated by at least three days within a two-week period.

Session one involved the participants being assessed for their body composition and exercise performance variables for all participants, including ${\dot{V}O}_{2\mathrm{peak}}$ and the individualized intensity associated with COP, which is close to Fatmax intensities [23,28]. Sessions two and three involved the participants being randomized to ingest either 2 g (4 × 500 mg capsules) of YM capsules or a placebo (PLC) empty capsules presented in similar color, size, and appearance. The YM capsules contained a standardized dried ground YM green leaves (Rio Trading Health Company, Brighton, UK), and all YM capsules were obtained from the same batch number (No. 17558), with approved content and safety. A certificate of analysis was obtained and included analyses of aspect, color, aroma, moisture, total and insoluble ash percentage, caffeine content (approximately 1.5%, minimum 0.4%), granulometry, but not the polyphenol content. Each YM capsule weighed approximately 575 mg (total weight of the four capsules 2.3 g), and a typical powder density within the capsule size used was 475–480 mg, the YM weight in the four capsules was approximately 2 g. Participants ingested all four capsules at once from a special weighted mug (292.1 g) with a <1% difference in its weight between treatments. A total of 500 mL of water was consumed over the duration of each testing visit as a standardized amount for all participants and to reduce the risk of dehydration, 300 mL with the supplement and 200 mL over the total remaining testing period. Participants were required to refrain from consuming caffeine for a minimum of seven days prior to the first testing visit and over the duration of the study. Participants also completed a three day (two week days and one weekend day) food diary with details about serving amounts for breakfast, lunch, dinner, snacks, and additional meals. A blank food diary and an example food diary were provided to each participant. Subjects were instructed on how to complete the food diary and asked to provide full details on all food, drinks, and snacks consumed both inside and outside the home including the portion size and the method of preparation. Participants were asked to replicate their recorded food dietary intake for each experimental test.

### 2.3. Baseline Assessments (Visit One)

Body composition was assessed using bioelectrical impedance scale, which also provides body weight, assessed to the nearest 0.1 kg (InBody version 720, Biospace, Seoul, Korea). Height was assessed to the nearest 0.5 cm using a Stadiometer (Seca scales, Hamburg, Germany).

The baseline exercise protocol involved the participants completing an incremental exercise test using an electronically braked cycle ergometer (Lode, Excalibur Sport, Groningen, The Netherlands). The saddle height and distance from the handlebar were recorded at the initial visit and re-applied in all subsequent visits. Participants began cycling at 25 watts for three minutes and work rate increased by 25 watts every three minutes until volitional exhaustion. ${\dot{V}O}_{2}$ and ${\dot{V}\mathrm{CO}}_{2}$ data were measured breath-by-breath using an Ultima (Medgraphics CPX Ultima, Medical Graphics Ltd., Gloucester, UK). Participants were fitted to wear a nose clip and breathe through a mouthpiece attached to a pneumotach. The volume and gas analyzers of the system were calibrated using a 3-L calibration pump and calibration gases (12%O_2_; 5%CO_2_) as per manufacturer’s instructions. Heart rate (HR) was recorded every minute using a HR monitor (FS1 Polar electro OY, Kempele, Finland). The rate of perceived exertion (RPE) was recorded during each stage using the (6–20) Borg scale [29].

The breath-by breath cardiorespiratory data of ${\dot{V}O}_{2}$ and ${\dot{V}\mathrm{CO}}_{2}$ were averaged over the last minute of each 3-min stage during the incremental test where the RER was <1 (below CHO saturation level). The data was also averaged for the last minute of every time point (5 × 30 min) at the 2-h rest and (6 × 5 min) during exercise the following two steady-state sessions. For each of the stages, EE (Kcal/min), FAO (g/min), CHO (g/min) were calculated using stoichiometric equations and corresponding energy metabolic equivalents, similarly to previous studies [22]. The COP intensity, which was used for the YM and PLC exercise trials, was determined for each individual, as the power output (W) and exercise intensity relative to ${\dot{V}O}_{2\mathrm{peak}}$ (% ${\dot{V}O}_{2\mathrm{peak}}$) corresponding to when the relative EE derived from CHO predominates (50% or higher) over that of FAO [24]. This intensity was used during the steady state exercise in two experimental trials (PLC and YM) for each participant.

${\dot{V}O}_{2\mathrm{peak}}$ was considered maximal when two of the three following conditions were met; a levelling off ${\dot{V}O}_{2\mathrm{peak}}$ with further increasing workloads (an increase of ≤2 mL/kg/min), a heart rate (HR) within 10 beats per minute of the age predicted maximum (220 bpm—age) and a respiratory exchange ratio (RER) of >1.05 (8). The last 15 s of the maximal exercise test were averaged to determine ${\dot{V}O}_{2\mathrm{peak}}$. Peak power (P_peak_) was calculated as the highest power output for the last completed exercise stage before test termination. If the stage was incomplete P_peak_ was calculated based on the completed time fraction of the final stage in seconds, and the power increment as previously described for a similar protocol [22].

### 2.4. Exercise Assessment and Supplementation (Visits Two and Three)

Participants received the supplement immediately before resting for 120 min in a semi-recumbent position during which they remained awake, while not talking and limited their movement. At the end of the resting period, participants were transitioned to a cycle ergometer where they cycled continuously for 30 min at a workload pre-determined from their COP calculated during the baseline visit. The cadence for the exercise protocol was self-selected to fall within a range of 60–80 RPM for the two supplementation trials. ${\dot{V}O}_{2}$, ${\dot{V}\mathrm{CO}}_{2}$ and HR data were collected continuously and RPE was assessed every 10 minutes throughout the exercise protocol.

### 2.5. Appetite and Mood State

Appetite and satiety were measured using a visual analogue 100 mm scale (VAS), which recorded perceived hunger, fullness, desire to eat, and prospective food consumption [30]. The scale was anchored at each end with the labels “not at all” (0 mm) and “extremely” (100 mm). Subjects marked a line through the scale between the two extremes of the symptoms being rated which they considered to indicate the degree of the subjective feeling being rated. VAS was completed prior to supplementation, every 30 min during the 120-min resting period, immediately post exercise (0 min), and 30 min post exercise.

Profile of mood state (POMS) questionnaire [31] was used to assess participants’ mood state (perceived alertness, focus, energy, fatigue, and concentration). Participants were asked to rate their perceived mood on a scale ranging between one and five for all five categories. POMS questionnaires were completed prior to supplementation, every 30-min during the 120-min resting period, immediately post exercise, and 30-min post exercise.

### 2.6. Data Processing, Analyses, and Statistics

All data are presented as means and standard deviations. FAO, CHO, EE, HR, POMS, and VAS were analyzed using two-way repeated measures ANOVA (YM × Time), with YM supplement as within factors, and six time points during 30-min for the exercise metabolic data (FAO, CHO, EE, HR) as between factors. Bonferroni post hoc test was applied to analyze the differences at each time point. For VAS and POMS the measured time points were analyzed using two-way ANOVA (Treatment × Time) for both rest condition at time points (0, 30, 60, 90, and 120 min), and exercise condition immediately before supplementation (0-min), immediately before exercise (120-min), immediately after exercise (150-min), and post 30-min recovery following exercise (180-min). The area under the curve (AUC) was calculated using trapezoidal method, and was compared between the treatments using a paired *t*-test. For all statistics SPSS IBM statistics V24 was used and the significance level was set at *p* < 0.05.

## 3. Results

The exercise power output at the cross-over point (COP) determined during the baseline assessment was 50.8 ± 22.3 W corresponding to a relative exercise intensity of 37.5 ± 8.0% ${\dot{V}O}_{2\mathrm{peak}}$. Peak data determined at baseline assessment were (${\dot{V}O}_{2\mathrm{peak}}$ = 38.3 ± 4.7 mL/kg/min, RPE_Peak_ = 19.7 ± 0.5, RER_Peak_ = 1.13 ± 0.06, P_Peak_ = 194.5 ± 11.4 W, HR_peak_ = 177.1 ± 11.5 BPM).

### 3.1. FAO, CHO, EE, and HR during Exercise

**Figure 1:**
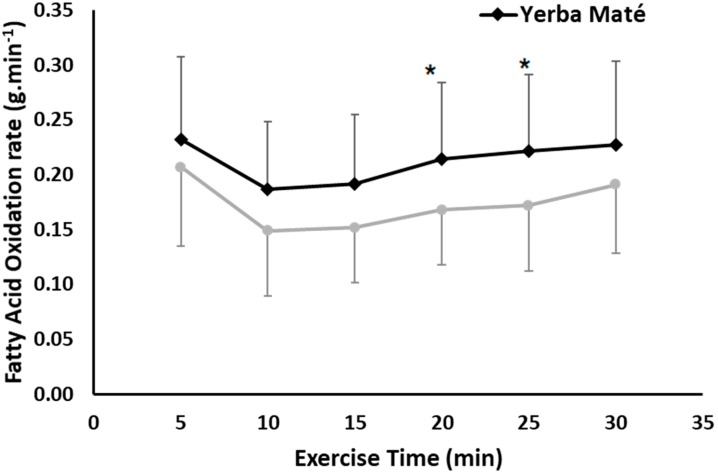
Fatty Acid Oxidation (FAO) during exercise in Yerba Mate (YM) vs. placebo (PLC). ANOVA treatment effect (*p* < 0.05). * Significantly higher in YM compared with PLC.

The YM treatment elicited significantly higher FAO compared with PLC during the 30-min steady-state exercise (ANOVA treatment effects, *p* = 0.037), (Figure 1). The difference was also affected by the time of exercise (ANOVA time effect *p* < 0.001). The highest FAO difference was after 20 min (0.21 ± 0.07 vs. 0.17 ± 0.05 g/min, *p* = 0.039), and 25 of exercise (0.22 ± 0.07 vs. 0.17 ± 0.06 g/min, *p* = 0.015) for YM compared with PLC (Figure 1). Calculating AUC showed a higher total AUC for FAO in YM than PLC (5.22 vs. 4.20, *p* < 0.001). However, there was no significant interaction between YM treatment and exercise time.

**Figure 2:**
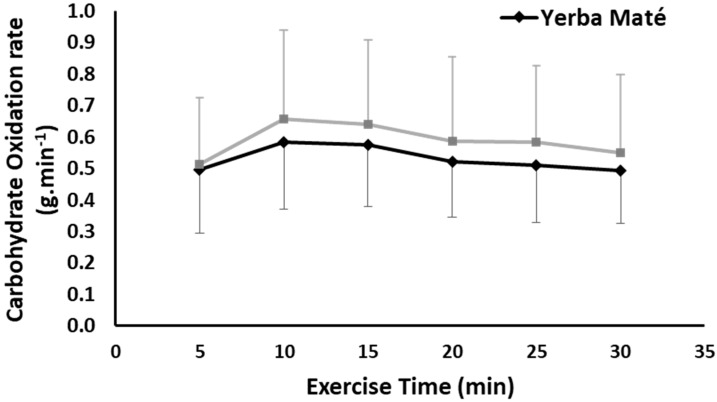
Carbohydrate oxidation during exercise in Yerba Mate (YM) vs. placebo (PLC), (*p* > 0.05).

CHO was not significantly different between the treatments, but there was a time effect (ANOVA time effects, *p* < 0.001) with the CHO initially increased and then decreased over exercise time in both treatments (Figure 2). No interaction for CHO between treatment and time was found. However, the total AUC for CHO was lower in YM than PLC (13.3 vs. 15.00, *p* < 0.001).

**Figure 3:**
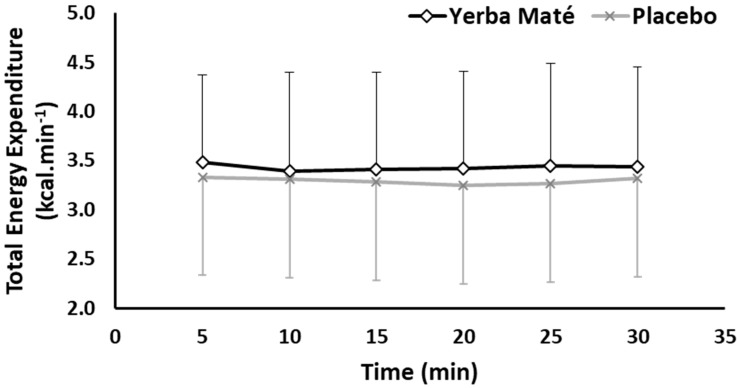
Total Energy expenditure during exercise in Yerba Mate (YM) vs. placebo (PLC).

Total Energy Expenditure (TEE) was neither significantly affected by the treatment (YM vs. PLC) nor the exercise time (Figure 3). However, the total AUC for TEE was higher (85.60 vs. 82.13, *p* < 0.001) in YM than PLC.

**Figure 4:**
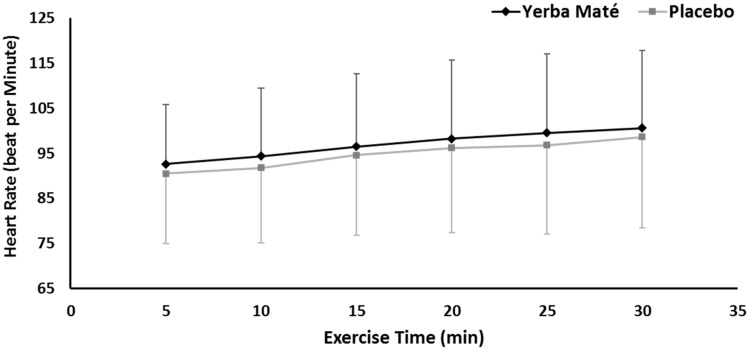
Heart rate response during exercise in Yerba Mate (YM) vs. placebo (PLC).

HR was not different between the treatments, but the time had significant effects (*p* < 0.001), (Figure 4). No interaction effects were found.

**Figure 5:**
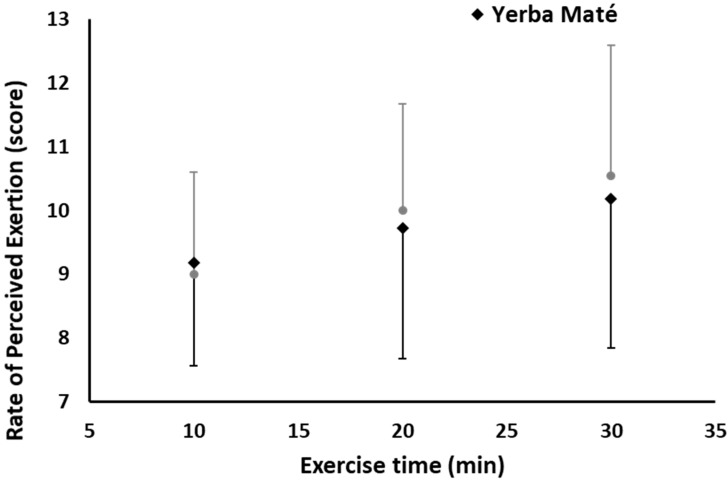
Rate of perceived exertion during exercise in Yerba Mate (YM) vs. placebo (PLC).

RPE, measured at three exercise time points, was also not affected between the treatments (YM vs. PLC) or exercise duration, nor was there any interaction effect between YM and exercise time (Figure 5).

### 3.2. Satiety Measures of VAS

There were significant treatment effects (YM vs. PLC) on VAS measures of Hunger (*p* = 0.019), Prospective eating (*p* = 0.022), and a trend towards a reduced desire to eat (*p* = 0.079) for exercise (Pre, immediately before, after, and 30 min post exercise). There was no YM effect on fullness score. The time effect was not significant for all VAS satiety measures, and there was no interaction between time and YM treatment (Table 1).

When analyzing the resting data only (immediately pre-ingestion 0 to 120 min rest duration), only prospective eating score was slightly reduced (*p* = 0.046) at YM treatment compared with PLC, but there were no significant YM treatment or rest time effects in any of the remaining VAS measures, nor were there any interaction effects (Table 1).

### 3.3. Mood State Measures of POMS

YM significantly affected POMS measures following exercise by an increased focus (*p* = 0.022), energy (*p* = 0.008) and concentration (*p* = 0.003), and a trend towards an increased alertness (*p* = 0.066) in the YM treatment compared with PLC (Table 2). No effects were found for fatigue scores. Exercise time had no effect on any of the POMS measures nor was there any interaction between treatment and time (Table 2)*.*

When analyzing the resting data only (immediately pre-ingestion 0 to 120 min rest duration) no significant effects were found for YM treatment, rest duration, nor were there any interaction effects (Table 2).

## 4. Discussion

In this experiment, YM increased FAO during prolonged steady-state exercise and induced positive psychomotor mood state and satiety during and after exercise without affecting the exercise RPE.

Augmented FAO was approximately 23% higher in YM compared with PLC during 30 min of steady-state low-to-moderate intensity exercise corresponding to individuals’ COP intensity (Figure 1). This increase is comparable with 24% increase found during low to moderate exercise intensities determined using an incremental protocol in our previous study [22]. The present study extended previous findings by determining COP individually, and demonstrated that YM ingestion is effective in enhancing the impact of FAO at the targeted COP exercise intensities. Targeting such intensities with exercise training enhances fat metabolism, and associated “fat-loss” metabolic health outcomes including increased insulin sensitivity [25], enhanced lipolysis and ability to oxidize lipids [32,33], and microvascular activity [26]. Therefore, YM augments such metabolic outcomes when combined with prolonged exercise at such given fat-loss intensities.

Previous studies in human participants have shown promising effects of YM ingestion on metabolic rate and RER acutely [21], and after 12 weeks of ingestion, on blood lipid metabolites in healthy obese participants [11,21]. However, these metabolic effects were only tested at rest. YM was also administered, with various metabolic efficacy, in various doses of ≈1 g of proprietary multi-ingredient thermogenic blends containing weight-loss ingredients such as YM and green tea extracts, caffeine anhydrous, guarana, yohimbine HCI, capsicum, ginger and bitter orange extracts, and other proprietary blends [16,22,34]. In two separate studies conducted previously, we tested the exercise-dependent effects on FAO at various intensities with 1 g YM [22] or when YM combined with a proprietary thermogenic blend of 1.5 g dose [15]. Both studies used mixed gender samples and showed an augmented FAO during low-to-moderate intensity exercise of 24% and 26% in YM compared with PLC. This is close to the 23% found for FAO in this cohort of female participants, using a higher ingestion dose of 2 g (Figure 1). The 38% ${\dot{V}O}_{2\mathrm{peak}}$ intensity used in this study is less than intensities (40–70%) [22] and 44% ${\dot{V}O}_{2\mathrm{peak}}$ [15] used in those two previous studies, but demonstrated almost equal % difference in FAO, which suggests that supplementing a higher dosage of 2 g could be more effective at higher intensities, and merits further investigation. Such intensity effects of the higher dosage used in the present study could also be attributed to the no significant difference found in the TEE (Figure 3). All three studies used sufficient amounts of resting time of 1–3 h following ingestion and prior to exercise, which is considered sufficient to induce the YM thermogenic effects at rest [21] and during exercise [22]. Other available studies to compare our findings with during exercise are limited to herbs which share some similar active ingredients to YM, especially green tea [4,5]. For example, Gahreman et al. (2015) [35] combined green tea with intermittent exercise and showed an increased FAO, plasma glycerol, and plasma catecholamines at rest and post exercise compared with placebo in healthy active female participants of similar characteristics to the present study. Another study by Hodgson et al. (2013) [36] found that a drink containing 1.2 g of green tea affected the metabolic profile (3-β-hydroxybutyrate, pyruvate, lactate and alanine concentrations) at rest and during 60 min of exercise at 56% ${\dot{V}O}_{2\mathrm{peak}}$ compared with placebo. However, both studies used higher intensities which promotes CHO metabolism, than the COP intensity of 38% ${\dot{V}O}_{2\mathrm{peak}}$ used in the present protocol.

It is likely that active YM thermogenic ingredients work in synergy to promote lipolysis and augment FAO during exercise. The metabolic effects include adrengenic effects and stimulated central nervous system associated with caffeine, anti-lipolytic, and hypocholesterolemic properties in chlorogenic acids (mono- and di-caffeolquinic acids) hydroxycinnamic acids (caffeic acid, quinic acid) and triterpenic saponins, and other minerals and vitamins [5]. Anti-oxidant compounds in YM such as flavonoids and polypheonols are common in other herbal teas and may affect nitric oxide levels, which have been shown to be effective in inducing vasodialatory effects [37] when combined with exercise [27,38]. Anti-oxidant compounds of YM have been recently attributed to accelerating muscle strength recovery 24 h after exercise, suggesting that YM favored the concentration of blood antioxidant compounds [39]. Therefore, YM active ingredients may have played a synergetic role in the metabolic effects found during exercise. However, further research is required to assess active ingredients of YM capsules and analyze their bioavailability following ingestion.

Favorable psychomotor effects on mood state and satiety are often expected outcomes of fat and weight-loss supplementation protocols. However, several negative side effects were reported for common thermogenic supplements containing caffeine including jitteriness, mood swings, and headache [40]. It is suggested that these effects can be reduced with YM ingested with other ingredients compared with caffeine [22,40]. The present study found an improvement in almost all measures of POMS and VAS (Table 1). In particular, there was an increased focus (*p* = 0.022), energy (*p* = 0.008) and concentration (*p* = 0.003) in the YM treatment compared with PLC, which was combined with no effects on fatigue scores. Interestingly, the RPE score was not different (Figure 5), suggesting that the positive psychomotor YM effects had no negative effects on the perception of effort and fatigue during exercise, which is important when considering adherence to prescribed exercise for weight loss and sport performance outcomes.

There was also a reduction in appetite VAS measures, especially for hunger (*p* = 0.019) and prospective eating (*p* = 0.022), (Table 1) following the YM ingestion compared with PLC. These YM appetite control effects are reported in humans for the first time, considering previous positive effects reported in animal models [13,14], and the recent report of appetite suppression following exercise in trained female participants [41]. The reduced VAS appetite scores are also in line with previous results found when YM was combined with other multi-ingredients before and after moderate exercise at Fatmax intensities at 43% ${\dot{V}O}_{2\mathrm{peak}}$, which is slightly higher than COP intensities of 38% ${\dot{V}O}_{2\mathrm{peak}}$ to the present COP intensities [22]. Nonetheless, it is suggested that irrespective of the intensity differences, exercise suppresses appetite hormones (GLP-1, PYY3-36, and acylated ghrelin) and VAS scores in trained women [41]. It has also been reported that exercise combined with satiety-inducing compounds is effective in reducing energy intake in active females [42]. This suggests that YM effects on satiety and mood state may be dependent on augmented metabolism during exercise. Perhaps, exercise combined with YM is most effective in appetite control and improved mood state after performing exercise, which is important for designing lifestyle interventions and weight-loss adherence.

HR response is a standard measurement for exercise intensity and training cardiovascular adaptations, but it was not significantly affected by YM ingestion (Figure 5). Cardiovascular benefits for YM consumption have been reported using more sensitive biomarkers such as detecting an increase in vascular endothelium-dependent vasorelaxing activity in rats [43,44]. Such microvascular measurements have been shown to be sensitive to the exercise-dependent effects in human lifestyle interventions [26]. Perhaps HR monitoring is insufficient to detect vascular responses associated with combining YM ingestion with exercise, and future research could determine more sensitive macro- and microvascular health effects associated with YM ingestion during exercise, especially when combined with detecting YM metabolic effects found in the present study.

Although there was not significance for all variables, the significant increase in FAO is mathematically accounted for by combined non-significant reductions in glucose oxidation and non-significant elevated TEE for YM (Figure 3). The estimates are approximately 0.35 kcal/min greater FAO, and 0.23 reduced CHO with 0.14 kcal/min greater TEE, and with a corresponding significant YM on the calculated total AUC for FAO, CHO, and TEE (*p* < 0.001). It is unclear whether there are additional YM effects on glucose and adipose tissue levels not determined in this study [45]. We used indirect calorimetry methodology to estimate metabolic variables, so perhaps future studies could use different techniques to estimate YM effects on different body compartments, tissues, and metabolites by including in vitro and muscle biopsy methods.

With respect to the gender-specific effects on metabolism, females’ FAO during exercise is known to be higher compared to men [46], possibly due to higher total body fat percentage, fitness level, and exercise modality [47]. The BF% data indicated all females who took part were at the lower BF% percentile, indicating that they were physically active, with higher ${\dot{V}O}_{2\mathrm{peak}}$ of 38 mL/kg/min, which is higher than 32 mL/kg/min measured for a mixed gender cohort in a previous similarly designed study [18]. The study’s participants completed their tests within the same week and reported to be within the same menstrual cycle phase. Some authors suggested that the luteal phase of the menstrual cycle is associated with increased lipid oxidation compared with the follicular phase [48], but no differences were reported by others during prolonged moderate exercise between luteal and follicular phases [46]. All participants within the present study repeated their assessments within the same phase of the cycle, so it is unlikely that menstrual cycle affected the metabolic variables. Nevertheless, gender differences’ effects on the combined YM and exercise-induced fat oxidation response needs further investigation.

## 5. Limitations

Although the positive effects of YM on fat-loss outcomes during exercise are found only in single trials, future research could test the longitudinal YM effectiveness, especially when combining regular exercise training with YM treatment. This is important for lifestyle disease prevention, given the effectiveness found for YM alone as treatment for human obesity, found in recent randomized controlled trials [10,11]. The present study used COP as an effective and a well-established “fat burning” exercise training intensity [24,32,33], but other established concepts in a similar domain, such as Fatmax intensity, can also be effective as we previously demonstrated [15]. The debate concerning a relevant exercise training intensity to maximize “fat-loss” is beyond the scope of this study, and has been sufficiently discussed elsewhere [23,24,28,32,33].

Most of the studies cited within the present study have attributed their YM ingestion effects to the several bioactive ingredients and only some of those studies have analyzed YM supplement (e.g., total phenolic compounds, caffeine, and saponins) for such ingredients as part of their protocol [39]. However, methodological differences in terms of YM supplement preparations and methods used should be considered for such comparisons. For example, the encapsulated ground green YM leaves used in this study is different from lyophilized YM extracts brewed and used elsewhere [39]. Also, the study did not specifically analyze the polyphenolic content of YM, and only relied on the supplier’s certificate of analysis for YM content, which makes it difficult to attribute findings to a single YM ingredient. Nonetheless, it is reasonable to infer synergetic effects of naturally-occurring YM ingredients but, from a biochemical point of view, performing phytochemical analysis on the supplement is important for future research. It would be also interesting to follow up with testing the YM active ingredients’ bioavailability post ingestion. Limited research has been conducted in humans prior to this study [10,11,22], which makes it difficult to confirm some parallels made with animal models when explaining YM mechanisms. Nonetheless, our findings in human participants extend previous knowledge and demonstrate novel applications of YM for human metabolic health.

## 6. Conclusions

Combining YM ingestion with exercise prescription at COP training intensities improves FAO, measures of satiety and mood state compared with exercise alone. The underlying mechanisms of such effects require further investigation. The combined exercise and YM effects on metabolic, psychomotor, and appetite control outcomes are essential for designing an optimized lifestyle prevention for metabolic health and exercise fat-loss outcomes. Future research should test the longitudinal effectiveness of YM and exercise for metabolic health outcomes.

## Figures and Tables

**Table 1:** Satiety results of Visual Analogue Scale (VAS) for Yerba Mate (YM) and placebo (PLC) treatment at different time points, at rest (0–120 min), exercise (120–150 min), and post exercise (180 min). Results displayed as Mean ± SD.

VAS Measures	Time (Min)	Score for YM	Score for PLC
Hunger	0	5.34 ± 2.62	4.76 ± 2.18
	30	5.01 ± 2.30	5.21 ± 2.74
	60	5.32 ± 2.43	5.80 ± 2.69
	90	5.85 ± 2.42	6.07 ± 2.23
	120	6.08 ± 2.16	5.83 ± 2.04
	150	5.89 ± 2.38	5.50 ± 2.29
	180	6.45 ± 2.52	6.88 ± 1.76
Fullness	0	2.06 ± 1.45	1.63 ± 1.16
	30	2.11 ± 1.37	1.73 ± 1.38
	60	1.55 ± 1.22	1.64 ± 1.36
	90	1.43 ± 1.05	1.67 ± 1.55
	120	1.55 ± 1.26	1.60 ± 1.27
	150	1.77 ± 2.17	1.46 ± 1.18
	180	1.94 ± 2.49	1.02 ± 0.96
Desire to Eat	0	5.45 ± 2.57	5.67 ± 2.08
	30	5.37 ± 2.25	6.10 ± 2.41
	60	5.96 ± 2.54	6.08 ± 2.49
	90	6.07 ± 2.39	6.54 ± 2.09
	120	5.76 ± 2.09	6.43 ± 1.89
	150	5.99 ± 2.70	5.74 ± 2.62
	180	6.34 ± 2.35	7.07 ± 1.95
Prospective Consumption	0	4.30 ± 1.90	4.65 ± 1.89
	30	4.47 ± 1.63	4.68 ± 1.86
	60	5.01 ± 2.02	4.80 ± 1.81
	90	4.97 ± 1.95	5.16 ± 1.62
	120	5.17 ± 1.96	5.36 ± 1.43
	150	5.21 ± 1.82	4.87 ± 2.17
	180	5.28 ± 1.59	5.64 ± 1.66

**Table 2:** Profile of Mood State (POMS) results for Yerba Mate (YM) and placebo (PLC) treatments at different time points, at rest (0–120 min), exercise (120–150 min), and post exercise (180 min). Results displayed as Mean ± SD.

POMS Measures	Time (Min)	Score for YM	Score for PLC
Alertness	0	3.18 ± 0.98	3.18 ± 1.17
	30	3.27 ± 0.79	2.82 ± 0.87
	60	3.36 ± 0.50	3.00 ± 1.00
	90	3.09 ± 0.83	2.73 ± 0.79
	120	3.18 ± 0.60	2.82 ± 0.87
	150	3.73 ± 0.79	3.73 ± 0.65
	180	3.36 ± 0.92	3.00 ± 1.00
Focus	0	3.45 ± 1.04	3.36 ± 1.21
	30	3.36 ± 1.03	2.82 ± 0.98
	60	3.18 ± 0.75	2.91 ± 1.14
	90	3.09 ± 0.70	2.82 ± 1.08
	120	3.18 ± 0.60	2.91 ± 0.70
	150	3.18 ± 0.60	3.45 ± 0.82
	180	2.82 ± 0.75	2.91 ± 0.94
Energy	0	3.00 ± 1.00	3.09 ± 0.94
	30	3.00 ± 1.10	2.82 ± 0.87
	60	3.00 ± 0.63	2.82 ± 0.87
	90	2.91 ± 0.70	2.91 ± 0.83
	120	2.82 ± 0.60	2.82 ± 0.75
	150	3.27 ± 0.47	3.73 ± 0.79
	180	2.64 ± 0.67	3.00 ± 0.89
Fatigue	0	2.73 ± 0.90	2.73 ± 1.01
	30	2.45 ± 0.82	2.91 ± 0.83
	60	2.55 ± 0.82	2.82 ± 0.60
	90	2.55 ± 0.93	2.82 ± 0.60
	120	2.64 ± 0.81	2.55 ± 0.69
	150	2.36 ± 0.81	2.18 ± 0.75
	180	2.64 ± 0.92	2.73 ± 0.79
Concentration	0	3.09 ± 1.04	3.27 ± 1.10
	30	3.00 ± 1.00	2.91 ± 1.14
	60	3.27 ± 0.79	2.91 ± 1.14
	90	2.91 ± 0.70	2.82 ± 1.08
	120	2.91 ± 0.70	2.73 ± 0.90
	150	3.18 ± 0.98	3.55 ± 0.93
	180	2.64 ± 0.81	2.82 ± 0.87
